# The Expanding Computational Toolbox for Engineering Microbial Phenotypes at the Genome Scale

**DOI:** 10.3390/microorganisms8122050

**Published:** 2020-12-21

**Authors:** Daniel Craig Zielinski, Arjun Patel, Bernhard O. Palsson

**Affiliations:** 1 Department of Bioengineering, University of California, San Diego, San Diego, CA 92093, USA; dczielin@ucsd.edu (D.C.Z.); arpatel@eng.ucsd.edu (A.P.); 2 Novo Nordisk Foundation Center for Biosustainability, Technical University of Denmark, 2800 Lyngby, Denmark

**Keywords:** synthetic biology, metabolic modeling, machine learning, metabolic engineering

## Abstract

Microbial strains are being engineered for an increasingly diverse array of applications, from chemical production to human health. While traditional engineering disciplines are driven by predictive design tools, these tools have been difficult to build for biological design due to the complexity of biological systems and many unknowns of their quantitative behavior. However, due to many recent advances, the gap between design in biology and other engineering fields is closing. In this work, we discuss promising areas of development of computational tools for engineering microbial strains. We define five frontiers of active research: (1) Constraint-based modeling and metabolic network reconstruction, (2) Kinetics and thermodynamic modeling, (3) Protein structure analysis, (4) Genome sequence analysis, and (5) Regulatory network analysis. Experimental and machine learning drivers have enabled these methods to improve by leaps and bounds in both scope and accuracy. Modern strain design projects will require these tools to be comprehensively applied to the entire cell and efficiently integrated within a single workflow. We expect that these frontiers, enabled by the ongoing revolution of big data science, will drive forward more advanced and powerful strain engineering strategies.

## 1. Introduction

Microbes have been engineered for a broad number of applications. As cell factories, cells have been designed to convert low-value substrates into valuable chemical products, including biofuels [1], commodity chemicals [2], bioactive compounds [3], and foods [4]. To benefit the environment, microbes have been engineered for bioremediation [5] and biosensing [6] of toxic compounds and pollutants. As engineered tools, microbes have been programmed using cell circuits to exhibit elaborate behaviors, from synchronized fluorescence [7] to hunting down tumors to deliver chemotherapeutics [8]. Finally, as cellular products, microbes themselves are increasingly of interest for probiotic and nutritional supplements [9].

**Figure 1:**
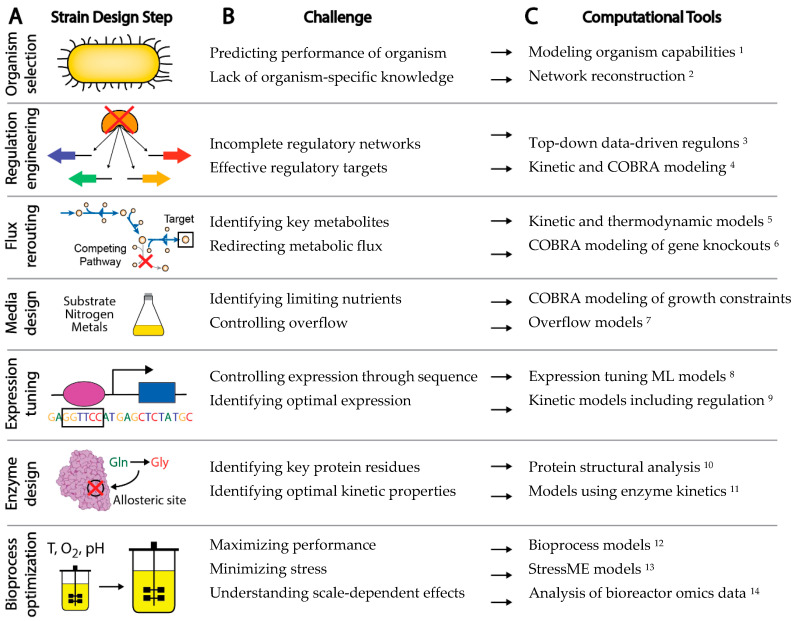
Challenges and computational solutions for a typical strain design workflow. (**A**) Typical experimental steps in the development of a new strain design. (**B**) Common challenges encountered at each strain design step. (**C**) Computational tools that may be used to meet the strain design challenges. Note that the design steps, challenges, and computational tools highlighted here are intended to be exemplative rather than comprehensive. ^1^, Modeling organism capabilities [27]; ^2^, Network reconstruction [18]; ^3^, Top-down data-driven regulons [28]; ^4^, Kinetic and COBRA modeling [26]; ^5^, Kinetic and thermodynamic models [29]; ^6^, COBRA modeling of gene knockouts [30]; ^7^, Overflow models [31]; ^8^, Expression tuning ML models [32]; ^9^, Kinetic models including regulation [33]; ^10^, Protein structural analysis [34]; ^11^, Models using enzyme kinetics [35]; ^12^, Bioprocess models [36]; ^13^, StressME models [37,38,39]; ^14^, Analysis of bioreactor omics data [40].

The experimental workflow to engineer a new microbial strain has a number of common steps, although the order may vary (Figure 1A) [10]. First, a background organism and strain is chosen for the application of interest. Genes may be knocked out, introduced, knocked down, or overexpressed for a variety of purposes, such as control of transcriptional regulation, redirection of metabolic flux to desired pathways, or removal of unwanted or wasteful processes. Bioprocess conditions can be optimized through control of various factors including media, feed rate, growth rate, pH, and temperature. Specific sequence variants can be introduced through rational design or selected through screens and adaptive laboratory evolution to control expression, alter enzyme activity, or remove regulatory sites from proteins. The typical strain design workflow thus requires a large number of decisions on how to improve strain behavior. Left to a trial and error approach, the complexity of biological systems makes efficient engineering of strains a daunting task.

To aid strain design efforts, computational tools have been integrated from various fields into the strain design workflow [11]. These tools offer the promise of restricting the experimental search space by either identifying modifications that are more likely to improve strain performance or proposing entirely new designs through mathematical modeling of cell behavior. However, many steps in the strain design process are still driven by rational approaches, rules of thumb, and extensive experimental screening and trial and error. Workflows driven purely by predictive tools would have the advantage of efficiency of execution through fewer experimental steps, reduced time, and ultimately improved performance through careful guidance toward an optimal desired phenotype. We describe two approaches that show promise as systematic tools for cell design: genetic circuits and genome-scale modeling.

One strategy for constructing synthetic strains has been to engineer desired behaviors through the use of genetic circuits [12]. The key concept is to carefully characterize and often mathematically model the behavior of a ‘circuit’, typically a small transcriptional regulatory network, to control a cell phenotype. As greater numbers of these small circuits are characterized, they begin to comprise a ‘parts list’ of available phenotypes from which an engineer can choose or can be assembled automatically by an algorithm [13]. Larger and larger circuits can then be constructed of well-characterized smaller circuits to engineer more complex phenotypes. This strategy has been employed for a number of promising applications [14,15].

Another successful paradigm for computational design of cells is genome-scale network modeling [16]. While genetic circuits approaches utilize highly controllable systems of limited scope, genome-scale models seek to predict cell phenotype by comprehensively modeling all known functions of the cell. As part of the Constraint-based Reconstruction and Analysis (COBRA) framework, genome-scale models of metabolism utilize a metabolic network reconstruction to predict metabolic phenotypes and analyze genome-scale datasets [17]. These models deal with the large scope of the system by utilizing the constraint-based modeling framework, which requires few parameters to generate predictions. The challenge of managing these large-scale models is achieved through community enforcement of rigid requirements, testing, and data standards [18,19]. Although these models were originally developed for metabolism, they have recently been extended to include transcription and translation machinery [20,21,22] and even further to whole-cell kinetic simulations [23].

Although computational methods have undoubtedly augmented rational strain design efforts, there are a number of challenges in a strain design workflow that still cannot be effectively addressed by existing computational tools [24] (Figure 1B). For example: (1) Organisms are often chosen for a strain design project due to historical knowledge and convenience, rather than fundamental benefits provided by the organism that could be calculated computationally *a priori*, (2) Gaps in gene annotation make choosing non-model organisms a risk, (3) The difficulty in accounting for enzyme kinetics makes the understanding of metabolic and allosteric regulation a challenge, (4) A lack of understanding of regulatory networks impedes the understanding and control of gene expression, and (5) Insufficient annotation of the organism genome makes it difficult to interpret the functional implications of sequence variation. Challenges such as these present major barriers to interpreting data and predicting strain phenotype.

**Figure 2:**
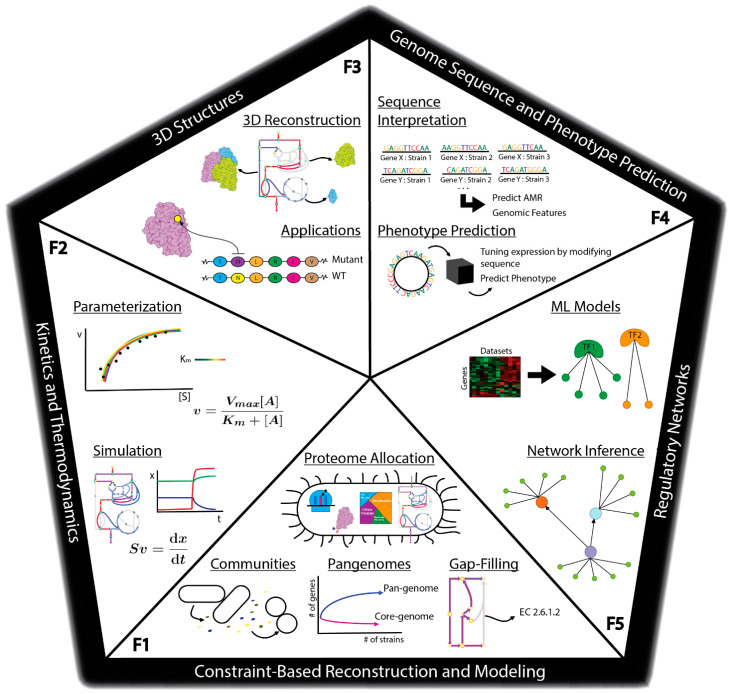
Overview of frontiers in the computational design of synthetic organisms. Frontier 1: Constraint-based Reconstruction and Modeling, consisting of tools for analyzing pan-genomes, microbial communities, gap-filling metabolic networks, and modeling proteome allocation. Frontier 2: Kinetics and Thermodynamics, consisting of tools for parameterizing and simulating kinetic and thermodynamic models. Parameterization can utilize the Michaelis-Menten equation where [A] is the substrate concentration, whereas simulation uses dynamic mass balance equations where S is the stoichiometric matrix. Frontier 3: 3D Structures, consisting of methods for the reconstruction of 3D metabolic networks with protein structural information and subsequent applications of these 3D reconstructions. Frontier 4: Genome Sequence and Phenotype Prediction, consisting of workflows for analyzing strain variations in genome sequence as well as building machine learning models based on genome sequence to predict strain phenotype. Frontier 5: Regulatory Networks, consisting of methods for the determination of transcriptional regulatory networks and subsequence models of gene expression and strain phenotype utilizing regulatory network information.

There are many methods currently being developed that may directly meet these challenges to enable fully predictive strain design workflows (Figure 1C). For example, advances in metabolic modeling could enable the optimization of bioprocess conditions or the identification of optimal expression levels of pathway genes [25,26]. However, these models are still in development and have not yet been shown to enable accurate predictions at scale. In this perspective, we describe five frontiers consisting of promising developments in computational strain design that may pave the way toward achieving comprehensive and integrated strain design workflows (Figure 2).

## 2. Frontier 1: Constraint-Based Reconstruction and Modeling

COBRA methods and tools have been used and refined for over 15 years in the field of systems biology [17,41,42,43]. Many current applications of constraint-based models answer biologically meaningful questions such as max growth rate, metabolite production, limiting nutrients, and gene essentiality. COBRA methods are also often used when little parameter and/or metabolite information is available given the size of the networks. These methods have predominantly been limited by annotation gaps in the underlying reconstructed networks [44]. COBRA models have also been expanded to include other cellular processes, such as transcription and translation, in order to increase the predictive capabilities of the models. Previous models did not have the capacity to accurately model suboptimal states, such as thermal or oxidative stress, nor could they properly describe how microbes interact within a community [24]. In the following sections, we describe recent computational advances that have benefitted COBRA models and methods.

### 2.1. Proteome Allocation Models

One of the main challenges for omics integration with M-models is that they only indirectly relate expression to metabolic fluxes. Metabolism and macromolecular expression (ME) models were developed to be multiscale models that explicitly include multiple cellular processes such as transcription, translation, and post-translational modifications. Early ME-models were able to compute optimal proteome compositions of a growing cell but suffered from significant model sizes and complexity, which has been addressed with the recent development of the COBRAme platform [20]. ME-models have since been produced for a variety of organisms and can now accurately predict overflow metabolism and cofactor/metal usage given media composition [45,46]. Additionally, ME-models have led to increased confidence in the selection of strain designs with robust growth-coupled production, in addition to having better byproduct secretion predictions than M-models [47,48]. Time course ME simulations are also possible and can predict substrate utilization hierarchy on mixed carbon source medium. These simulations compute distinct proteome compositions over time [49]. Prediction capabilities for ME-models have also expanded to include suboptimal states such as stress and mitigation responses. These ‘StressME’ models are able to more accurately model the cell’s unused proteome that acts as a hedging mechanism for preparedness functions under low pH, thermal, and oxidative stress [37,38,39].

Unfortunately, developing a ME-model requires a deep understanding of the organism’s cellular machinery. For organisms that lack the required annotations, less complex alternatives exist. MOMENT, or Metabolic Modeling with Enzyme Kinetics, was first developed to better predict metabolic fluxes and growth rates by using enzyme turnover rates and molecular weights [50]. GEMs can also be integrated with enzyme constraints using kinetic and omics data, which is also known as GECKO. Since GECKO does not require detailed knowledge about every step in protein synthesis its been applied to other organisms such as the eukaryal *Saccharomyces cerevisiae* [51,52]. Unlike GECKO, MOMENT does not allow for the direct incorporation of measured enzyme concentrations. Short MOMENT or sMOMENT was recently developed to allow for this type of integration while also greatly reducing the size and complexity of the original MOMENT model [53].

### 2.2. Communities

Modeling communities or co-cultures are important for healthcare and biotech applications since many times strains are paired based on metabolic coupling. For example, pairing phototrophs with heterotrophs is a promising prospect for sustainable biotechnology, since it enables heterotrophs like *Escherichia. coli* to grow in minimal media devoid of organic carbon sources. Community metabolic models (CM-models) can be constructed for co-cultures to aid in this strain selection. By modeling and simulating various synthetic microbial co-cultures, researchers are able to identify optimal pairs that produce the most active community [54]. In some cases, dynamic community metabolic models can be generated for co-cultures using each organism’s genome-scale metabolic network. These dynamic simulations are able to predict metabolite concentration profiles for the community as well metabolic exchange flux profiles for individual organisms [55]. Along a similar vein, communities are able to partition metabolic functions among community members like in cases of auxotrophy. These specialized pairings are capable of improving product yield and accomplishing more complex tasks as compared to a single strain [56]. The OptAux algorithm was created to aid in designing auxotrophic strains that need a metabolite cross-feeding co-culture [57].

### 2.3. Pangenomes and Multistrain Models

Reconstructing GEMs for multiple strains across a single species has enabled a systems-level approach to study and characterize the pan-metabolic capabilities of the species. Pangenomic studies have been accomplished for a wide range of species from *Staphylococcus aureus* to *Klebsiella pneumoniae* [58,59,60,61,62,63]. Integration of genomics, phenomics, transcriptomics, and genome-scale modeling for seven commonly used *E. coli* strains linked molecular features to strain-specific phenotypes. The integrated models showed that certain strains are better suited to produce specific compounds or phenotypes, which has implications for strain selection when choosing a platform strain for microbial engineering [27].

### 2.4. Gap filling, Discovery, and Annotation

In order to more accurately predict an organism’s phenotype, there needs to be a more complete genome annotation to better understand the organism’s capabilities. Even in *E. coli*, one of the best-studied model organisms, 35% of its genome lacks functional annotations, with many of these genes being experimentally linked to phenotypes [64]. Recently, there has been a multitude of computational tools that have taken strides in elucidating the possible functions of these genes.

Transposable elements (TEs) are of high interest when engineering a strain due to the deleterious effects they can have if uncontrolled. In platform strains, TEs or insertion sequences (ISs) are often removed in order to preserve the intended genomic content. Issues arise if the organism of interest is poorly characterized and the TEs are not known, however, new machine learning algorithms that utilize genome sequence are able to identify the TEs and ISs in both eukaryotic and prokaryotic species [65,66].

Gap-filling has been commonly used for reconstructing genome-scale metabolic models but over the past few years has quickly advanced in coordination with the recent developments in machine learning. Current methods such as DeepEC are able to predict enzyme commission numbers based on genome sequence with high accuracy [67]. Additionally, machine learning algorithms that use genome sequence can predict systems-wide enzyme promiscuity or candidate genes/enzymes for orphan reactions [68,69]. By filling these annotation gaps and identifying possible cases of promiscuity in microbial networks, researchers will be able to achieve more accurate and comprehensive predictions.

## 3. Frontier 2: Kinetics and Thermodynamics

Kinetic modeling of metabolism is a field with a long history dating back to the original work understanding enzyme kinetics at the beginning of the 20th century. These ODE-based models offer the promise of mechanistically accounting for the saturation and regulatory state of every enzyme, providing a direct representation of the mechanisms underlying cellular homeostasis [35]. Metabolic control analysis, rooted in steady-state analysis of a kinetic model, paved the way for quantitative analysis of metabolic networks in the early days of metabolic engineering [70]. Sharing much of the same underlying theory, constraint-based thermodynamic models calculate the energetic driving forces underlying metabolic fluxes and can be used to determine physiological constraints on reaction reversibility in the metabolic network [29,71,72,73,74]. Multi-scale models accounting for kinetics and thermodynamics have begun to emerge as well [75,76,77], as discussed in the earlier section on Proteome Allocation Models. The development of kinetic and thermodynamic models has been hampered by the difficulty in acquiring the necessary parameters and validating model behavior at large-scale [78]. Furthermore, the complexity of these models, need for accounting for parameter uncertainty, and additional confounding factors such as numerical instability in kinetic models or constraint infeasibilities in thermodynamic models, substantially increase computational requirements for large-scale modeling. However, recent advances in parameterization and simulation of kinetic and thermodynamic models promise to greatly expand the scope and accuracy of these models.

### 3.1. Parameterization

The critical step in the construction of a kinetic or thermodynamic model is specifying the values of the necessary parameters. This effort is complicated by the lack of required data which leaves these models largely underdetermined. Algorithms must be developed to fit parameters to available data and account for parameter uncertainty.

For parameterizing kinetic models, a number of approaches are now available: (1) Systems-level fitting, where all parameters of the model are fit simultaneously to systems-level data such as metabolomics and fluxomics [79,80], (2) estimation of kinetic parameters directly from in vivo data without the need of a model [81,82], (3) machine learning to estimate kinetic parameters [83], and (4) bottom-up reconstruction of kinetics on an enzyme by enzyme basis [84]. Similarly, methods to account for parameter uncertainty have advanced through powerful algorithms [85]. Thus, the ‘kinetome’, a genome-scale collection of the kinetic properties of metabolic enzymes, may soon be within reach [86].

In thermodynamic models, the majority of work has focused on estimating the standard Gibbs free energy of reaction, dG^0^_r_, which can be readily converted to the reaction equilibrium constant K_eq_. Experimentally, reaction equilibria are directly measured under a variety of biologically relevant experimental conditions, such as pH, T, IS, and magnesium concentration. To estimate the equilibrium properties of reactions lacking experimental data, the most popular approach has been the group-contribution family of methods [87,88], which has led to software such as eQuilibrator [89]. The ability to correct these estimates accurately for pH [90] and temperature [91] have since been added. However, there are a number of inherent flaws in group-contribution as an estimator, including fundamental limitations of the underlying additivity assumptions [92]. Methods for estimating compound Gibbs energies based on direct quantum chemistry predictions are a promising alternative [93].

### 3.2. Simulation

Kinetic models also present substantial computational challenges in simulation and analysis. Numerous issues including model stiffness due to poor conditioning, dynamic instability, and complex dynamic properties require sophisticated tools to manage dealing with kinetic models effectively. A number of software packages have emerged to meet this challenge [94,95,96]. Additionally, specialized methods for dealing with large-scale kinetic models have emerged by necessity [23,97,98].

Simulation of the thermodynamic properties of a metabolic network faces a distinct set of challenges. Thermodynamic simulation at the genome-scale, through constraint-based methods such as thermodynamic flux balance analysis (tFBA) [99] and network-embedded thermodynamics (NET) [29], must carefully account for uncertainty in thermodynamic parameters and metabolic concentration constraints. Thermodynamic optimization algorithms, such as tFBA, often involve a mix of integer and linear constraints, and MILP, MIQP, and MINLP algorithms may compute slowly at the genome-scale without efficient solving approaches. Integration of thermodynamic constraints with other biophysical constraints presents further complications due to the non-convexity of the resulting space [100].

## 4. Frontier 3: 3D Structures

Currently, structures are commonly used for analyzing observed sequence variants or identifying functional sites of a protein for targeted engineering. While structure-guided enzyme design remains a promising application, it is incredibly complex and often coupled to large experimental screens [101,102,103]. Software has been developed to aid in enzyme design, such as the Iterative Protein Redesign and Optimization (IPRO) method, but incorporating enzyme design into a strain design workflow remains difficult due to the required expertise and experimental validation required [104]. On the other hand, integrating protein structures into systems biology has shown promise as a more accessible addition to strain design workflows, but remains a challenge due to differences between the fields causing a steep learning curve. Here, we highlight some of the advances that have lowered the learning curve for using and integrating structures data with systems biology approaches.

### 4.1. 3D Reconstruction

GEMs have now been expanded to include protein structural information, which has enabled comparative structural proteome analysis between strains and organisms. These GEMs with protein structures (GEM-PROs) allow for a direct mapping of gene to protein structure to phenotype [34,105]. Software has been developed to aid with the construction of high-quality GEM-PROs and to visualize/annotate structures. The pipeline and software, ssbio, is available for use on GitHub (http://github.com/SBRG/ssbio) and is implemented in Python [106]. For cases where a protein is poorly characterized and the necessary mapping fails to exist, homology modeling or tools such as I-TASSER can be used to predict 3D structures and structure-based functional annotations [107]. AlphaFold was also recently announced as the best protein folding solution at the Critical Assessment of protein Structure Prediction (CASP)-14 competition [108]. CASP was founded in 1994 with the goal to establish the state of the art in protein structure prediction based, and AlphaFold 2 has recently achieved predictions competitive with results obtained from experimental methods, something that was once thought to be impossible.

### 4.2. Applications

Structural information is often used for 3D mutational mapping or visualization but has more capabilities when used in an integrated workflow. For example, GEM-PROs for multiple strains of the same species enables the comparison of sequence variants among conserved genes [105]. Additionally, combining machine learning approaches and 3D structural mutational mapping has identified genetic signatures of antimicrobial resistance evolution to multiple antibiotics in *M. tuberculosis* [109]. Brunk et al. developed a multiscale workflow to better understand the roles and mechanisms of protein post-translational modifications (PTMs) due to the challenges they present in engineering organisms and their interference with drug action [110]. This workflow incorporates genome-scale modeling, genome editing, and molecular enzyme assays to identify specific roles of PTMs and how they regulate cell phenotype [110]. Well-established software incorporating three-dimensional structural information also exists today. Amber is a package suite of computer programs and has been in development for over 40 years. Amber simulates molecular dynamics for proteins and other biomolecules using structural information and molecular mechanical force fields [111,112].

## 5. Frontier 4: Genome Sequence and Phenotype Prediction

The genome sequence lies at the heart of a strain design workflow. Heterologous genes must either be added via plasmids with established expression behavior or integrated into the chromosome, and the behavior of these genes depends on the sequence of both coding and non-coding regions of the genes. Further, mutations occur in any mutagenesis or adaptive laboratory evolution strain that control phenotype. Strain design projects may choose between different strains of a species, and the sequence variations between these strains may lead to diverse differences in behavior. Quantifying the sum of sequence factors to predict strain phenotype such as gene expression is an active area of research. Two related strain design tasks utilize the genome sequence: (1) analysis of observed sequence variation data and (2) prediction of phenotype based on genome sequence.

### 5.1. Sequence Interpretation

A combination of natural, selected, or randomized sequence variants of different genes will typically be observed or generated throughout a typical strain design project. Interpreting the effect of these mutations presents a significant challenge. Natural variants occurring across strains of a species have been analyzed to understand phenotypes related to antimicrobial resistance [109,113,114,115]. Machine learning models can be trained on a phenotype of interest, and key variables extracted to understand which genetic features are important for determining the phenotype [116]. A well-established example of this type of analysis is the identification of protein-DNA binding motifs, for example by the MEME suite [117]. Sequence variants occurring in the course of adaptive laboratory evolution have also been collected [118], and analyzed [119]. This type of sequence analysis is possible due to the recent development of data structures for contextualizing multiple data types within the genome [120]. Further follow up is required to understand the mechanistic basis establishing the relationship between these genetic features and the phenotype.

### 5.2. Phenotype Prediction

In addition to analysis of observed mutations, the genome sequence can also be used to directly predict strain phenotypes. The genome sequence directly affects the function of the protein product, the mRNA transcription rate, and the protein translation rate of a transcript, termed the translation efficiency. Not surprisingly, models capturing these effects are of great interest. While classically, the assignment of function to a new gene has been done primarily through sequence homology, more sophisticated methods based on machine learning are being developed that have the potential to better capture the sequence-function relationship [67]. Similarly, a number of machine learning and mechanistic models of methods predicting mRNA expression [121] and translation efficiency [122] from promoter and transcript sequence, respectively, have been developed. Other methods predict protein expression directly from sequence, integrating both transcription and translation effects [123]. These methods often are trained on large assays based on synthetic libraries of sequence variants. A key challenge is to achieve general applicability of these models in new experimental conditions and strain backgrounds.

## 6. Frontier 5: Regulatory Networks

Transcriptional regulatory networks (TRNs) for multiple organisms have been studied rigorously and have made significant advancements with the expansion of ChIP-seq data. Even with this deluge of experimental data, many questions still remain, even for *E. coli* with one of the best characterized TRNs. Computational approaches combining experimental datasets with machine learning methods have shown promise in further elucidating the underlying regulatory network, even from old low-resolution datasets. These new approaches and insights have opened the door for more accurately integrating metabolic modeling with regulation, a long time goal in the field of computational systems biology. The following will discuss these methods and their implications.

### 6.1. Regulatory Network Machine Learning Models

A few methods have been developed for further elucidating an organism’s TRN using machine learning. Applying independent component analysis (ICA), an unsupervised machine learning algorithm, to diverse transcriptomics compendia reveal statistically independent signals that modulate the expression of genes. In *E. coli,* 66% of the 92 identified signals represent the effects of transcriptional regulators, whereas 27% represent biological or genetic explanations. ICA decomposition has also proven effective for other organisms, such as *B. subtilis, and S. aureus* [28,59,124]. Models for these organisms and more information on the method can be found in the iModulon database (iModulonDB.org) [28,125]. Another method, Transcriptional Regulatory Network Analysis, or TReNA, combines TF/target gene correlations and TF DNA binding information to create gene regulatory predictions. The resulting predictions approach mechanistic accuracy when using high-resolution data collection techniques like single-cell RNA-seq and ChIP-seq, but bulk mRNA data and low-resolution techniques still provide a coarse-grained result. TReNA thus enables researchers to work along this spectrum [126]. While these processes utilize transcriptomics to predict regulators, algorithms also exist to predict gene expression as a function of transcription factor expression levels [127]. Probabilistic regulation of metabolism, or PROM, is a method for integrating TRNs and metabolic models. PROM uses conditional probabilities to represent gene states and gene-TF interactions, as well as FBA for modeling the metabolic network. The method was one of the first to enable automated integration of transcriptional and metabolic networks using high-throughput datasets [128].

### 6.2. Network Inference

Being able to predict network structure and identify the roles of specific vs. global regulators is necessary for a more complete understanding of how gene expression changes under varying sample conditions. Rustad et al. created a transcription factor overexpression (TFOE) library by cloning and overexpressing 206 TFs in *M. tuberculosis*. The resulting strains were used to identify sets of genes affected by TFOE and assembled into a global transcriptional map. The TFOE regulatory map identifies potential regulators of gene sets and was used by Rustad et al. to predict and validate the phenotype of a regulator affecting the susceptibility of isoniazid, an antibiotic used to treat tuberculosis [129]. Kochanowski et al. developed computational methods to identify global vs. specific transcriptional regulation based on promoter activity and to discern metabolites that serve as potential transcriptional regulators. The simple mathematical models use high-throughput measurements of metabolite concentrations and promoter activity. A model of *E. coli* central carbon metabolism showed that 90% of expression changes are due to a few regulatory metabolites (F1P, FBP, and cAMP) and global TFs (Crp and Cra) [130].

## 7. Drivers of Advances in Computational Tools for Strain Design

The improvements in both scope and accuracy of the computational tools discussed here have been driven by a combination of experimental and machine learning advances. The availability of large-scale datasets that enable comprehensive modeling has exploded. To list a few examples: (1) Continued determination of experimentally-determined protein 3D structures has empowered the rapid development of homology modeling of protein structures, (2) Falling sequencing costs and improved sequencing quality have resulted in a rapid increase of the number, assembly quality, and annotation quality of available microbial genomes, with similar increases to other sequence-based omics types, such as RNA-seq and ribo-seq, and (3) Improved quantitative proteomics datasets have enabled the large-scale estimation of in vivo enzyme turnover rates [81], greatly empowering kinetic and proteome-allocation modeling approaches. Alongside this increased availability of data, machine learning methods to effectively utilize this experimental data to parameterize biological models have greatly improved in recent years. In addition to machine learning algorithms, any expert in machine learning knows the importance of data curation and data cleaning to successful model development. For this reason, the growth and maintenance of highly curated organism-specific knowledgebases, such as RegulonDB and EcoCyc for *E.* coli, has been critical to providing a source of reliable data for many computational tools [131,132]. Thus, as these companion fields continue to evolve, it is expected that the accuracy of the computational tools built upon these data and algorithms will continue to rapidly improve.

## 8. Outlook for Synthetic Genome Design

**Figure 3:**
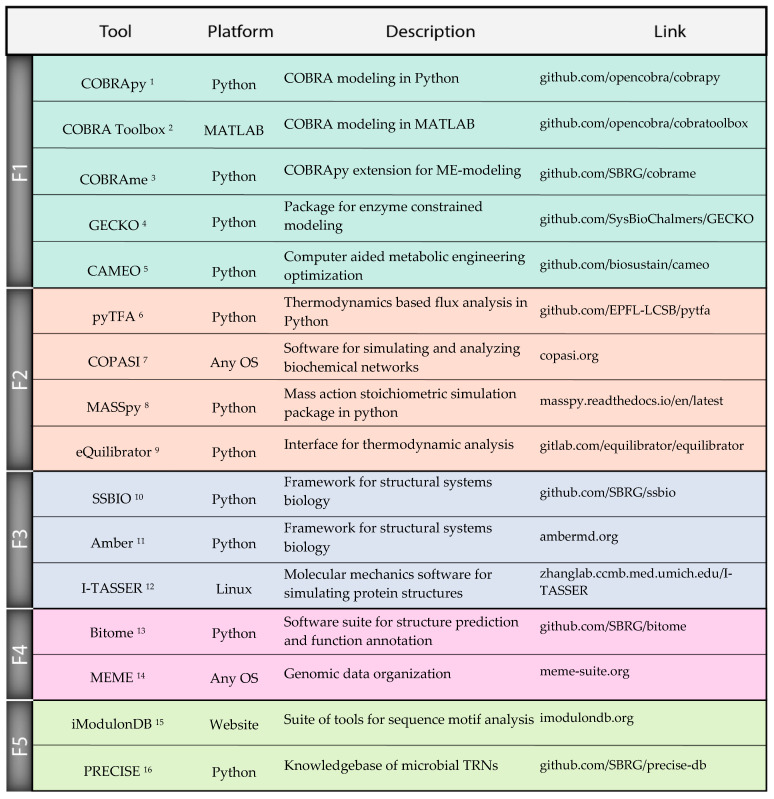
A selection of actively maintained software for computational design and analysis of microbial phenotypes. We focus on Python tools due to the popularity of the language as well as potential for integration in a single strain design workflow, but also include important packages in other languages and standalone applications. Frontier 1: Packages for constraint-based reconstruction and modeling, proteome allocation modeling, and strain design optimization. Frontier 2: Kinetics and thermodynamics packages for model parameterization, simulation, and thermodynamics constrained modeling. Frontier 3: Software for annotating and visualizing structures as well as integrating 3D structural information with systems biology approaches. Frontier 4: Python package for storing, organizing, and analyzing genome sequences. Frontier 5: Online knowledgebase and software for determining transcriptional regulatory networks using ICA decomposition methods. ^1^ COBRApy [42]; ^2^ COBRA Toolbox [17]; ^3^ COBRAme [20]; ^4^ GECKO [52]; ^5^ CAMEO [43]; ^6^ pyTFA [95]; ^7^ COPASI [96]; ^8^ MASSpy [94]; ^9^ eQuilibrator [89]; ^10^ SSBIO [106]; ^11^ Amber [111]; ^12^ I-TASSER [107]; ^13^ Bitome [120]; ^14^ MEME [117]; ^15^ iModulonDB [125]; ^16^ PRECISE [28].

Although there has been substantial progress in each of the individual fields discussed above, there are additional challenges with integrating these tools into an effective strain design workflow. Workflow: While we discussed many tools as they relate to individual strain design tasks, these tasks must be synthesized into a coherent end-to-end design workflow. The decisions of the order of operations in the development of a strain could greatly benefit from computational predictions, but much work is yet to be done to identify a strain design workflow that maximizes efficiency and minimizes cost and risk. Expertise: Any workflow that integrates many different computational tools will require domain expertise in each tool to decide details of implementation, from parameters to valid use cases. Thus, strain designers will be required to have broad computational skillsets that exceed what is taught by most current training programs. Software: The practical difficulty of implementing many separate computational tools can become a substantial burden, spanning various details from licensing issues to file formats. However, the number of software packages enabling these workflows continues to increase, and we mention many examples in this work (Figure 3). Thanks to these efforts, finding compatible tools for easily integrated workflows is becoming easier. Validation: Tools must be validated to clearly established accuracy metrics under physiological conditions. Validation of tools on individual datasets, for example on a single wild type strain background, is likely to be insufficient as the strain is engineered further from the wild type. To meet these challenges, it is critical to take a systematic approach that includes dedicated training, effective documentation of tools, and extensive validation of tools in real applications. There will be a significant challenge reaching a standard where strain design researchers can effectively conduct analyses and understand results from multiple tools across a typical workflow.

The field is nearing an important milestone in synthetic biology, that of the comprehensive and computationally-driven strain design workflow. We may soon enter an era of ‘computational genome design’, where rational approaches finally give way to biological design algorithms dominated by computational predictions. Thus, one of the early promises of the field of systems biology may finally be nearing its realization. The practical applications of such a cell design workflow are endless, from the chemical industry to the environment to human health.
